# Monitoring Liquid Slugs Using Distributed Acoustic Sensing and an Air Gun

**DOI:** 10.3390/s26041278

**Published:** 2026-02-16

**Authors:** Hyojeong Seo, Erasmus Mensah, Caio Morais De Almeida, Amy Amudzi-Deku, Smith Leggett

**Affiliations:** Bob L. Herd Department of Petroleum Engineering, Texas Tech University, Lubbock, TX 79409, USA; hyojseo@ttu.edu (H.S.); eramensa@ttu.edu (E.M.); cmoraisd@ttu.edu (C.M.D.A.); amyamudzideku9@gmail.com (A.A.-D.)

**Keywords:** distributed acoustic sensing, DAS, liquid slug tracking, riser systems, fluid level guns

## Abstract

Distributed acoustic sensing sends laser pulses along a fiber optic cable and analyzes the backscattered light to identify acoustic signals along the entire fiber. Liquid slugs were produced in a 427 m vertical test well using surface-controlled gas lift valves. To enhance DAS monitoring, pressure pulses were induced by multiple acoustic shots from a fluid level gun. Visualization of the responses through frequency band energy plots and unfiltered phase shift measurements permitted tracking slug movement and estimating parameters such as velocity, location, and body length. The results demonstrate that DAS stimulated with acoustic pulses can effectively track liquid slugs in real-time. We observe that relying solely on flow-induced noise in multiphase flow environments may not provide sufficient signal strength for slug detection. Applications include real-time detection of liquid slugs for improved well monitoring and flow management.

## 1. Introduction

Liquid slug production is a well-known flow assurance challenge in multiphase pipeline-riser systems and wells. Many wells and production systems have complex trajectories, including horizontal, deviated, and vertical sections. Liquids can accumulate in the production string under certain conditions, particularly when the well trajectory changes from horizontal to vertical. Once enough liquid accumulates, it blocks the gas flow, resulting in a pressure build up until the slug is rapidly accelerated upward. Slugging may occur due to several instability mechanisms, such as terrain-induced liquid accumulation, riser hydrodynamic instability, and gas lift flow instabilities. Ref. [1] analyzed the development of density wave oscillations in gas lift wells, while ref. [2] proposed a low-order model and active choke control strategy for mitigating severe slugging. Ref. [3] characterized the severe slugging cycle in flowline riser systems, and ref. [4] identified operating conditions that lead to unstable oscillations in gas lifted wells using distributed parameter modeling. Terrain induced slugging has been widely investigated in pipeline riser configurations operated under near constant separator pressure. Refs. [5,6] performed foundational air–water experiments that documented the complete severe slugging cycle. Several predictive models have also been developed. Ref. [5] introduced a mechanistic transient model, ref. [7] extended these predictions using a distributed parameter framework, and ref. [8] proposed stability criteria defining the operating boundaries for severe slugging. The vibration and dynamic loads induced by high-velocity liquid slugs can cause downhole and surface equipment failures, especially at bends, tees, and other points where the flow abruptly changes direction [9,10].

Detecting and predicting transient well conditions at an early stage significantly influences well control strategies, production stability, and risk management [11]. This enables operators to respond appropriately and promptly [12]. Operators would benefit from sensing technologies that monitor and predict severe liquid slug production. In this work, we aim to demonstrate a liquid slug detection method under field-scale operating conditions.

Fiber optic sensing technology has evolved through advances in interferometry, enhanced backscatter fibers, and coating technologies for harsh environments [13]. In the oil and gas industry, distributed fiber optic sensing has been increasingly used for real-time well and pipeline monitoring across all phases of operation [14]. There are three primary types of distributed fiber optic sensing. Distributed Temperature Sensing (DTS), Distributed Strain Sensing (DSS), and Distributed Acoustic Sensing (DAS). DAS can achieve fiber lengths up to 25 km with a spatial resolution of 2 m, as demonstrated by Wait and Hartog [15]. This technology is widely applied for wellbore diagnostics, such as flow characterization [16], zonal production profiling [17], and hydraulic fracture injection allocation [18].

Recent studies have extended DAS to analyze two-phase liquid–gas flows, including slug flow. In one study, researchers used DAS to capture transient borehole flow velocities from thermal slugging signals [19]. Video-based approaches have also been used to estimate parameters such as Taylor bubble velocity and frequency; however, such methods are often not practical for field deployment [20,21]. Another study utilized DAS to estimate velocity and identify patterns in two-phase gas–liquid slug flow using f-k transform and distributed cross-correlation [22]. The f-k transform represents the DAS signal as a function of wavenumber and frequency. Ref. [20] presented a different workflow and defined slug-related parameters including unit length, body length, and film region length.

Several of the aforementioned studies rely on experimental setups where the fiber is wrapped around the outside circumference of the flow conduit. While this setup enhanced the signal-to-noise ratio, it presents several limitations: difficulty of installation in real wells due to spatial, pressure, and temperature constraints; high installation costs and maintenance challenges; and incompatibility with typical field geometries where fibers are placed in the annulus. To address the signal-to-noise problem with fiber run axially along the outside of the flow conduit, we use a fluid level gun installed at the wellhead to generate a strong acoustic pulse within the production tubing.

Acoustic shots are a widely used method to diagnose a downhole phenomenon from surface. A sound wave is reflected at the liquid surface in the well, and the fluid level can be determined from the reflection time and velocity of the wave. Applications include detecting fluid levels in artificially lifted wells, identifying the location of holes in tubing or completion equipment, and tracking plunger velocities [23,24,25]. In this study, we assess the ability of DAS to detect these acoustic pulses and evaluate the system’s capability for distributed liquid-level detection. In contrast to well-controlled laboratory environments, by using DAS with applied acoustic pulses, our experiment demonstrates the ability to detect and track key slug features, such as location, velocity, and length under noisy field-scale conditions.

## 2. Materials and Methods

**Figure 1:**
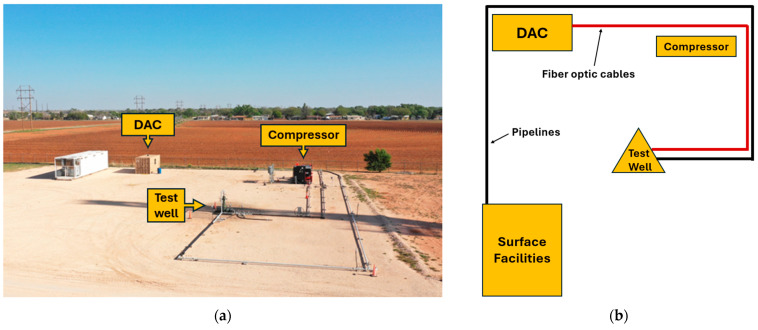
(**a**) Overview of the OTC. (**b**) Corresponding schematic diagram showing the key components of the setup.

We conducted liquid-slug tracking experiments at the Oilfield Technology Center (OTC) at Texas Tech University. Figure 1 provides a picture and schematic of the experimental site. The site includes a vertical test well, a pump for liquid injection into the wellbore, a compressor for gas injection into the wellbore, and a data acquisition container (DAC). The wellbore produces to a separation facility located approximately 274 m away. The facility includes a 0.76 m by 3 m three-phase separator and a 48-m^3^ capacity water tank for liquid storage. The gas line from the separator is connected to the compressor’s suction. This layout enabled controlled liquid and gas injection and real-time DAS data acquisition during slug formation and production.

**Figure 2:**
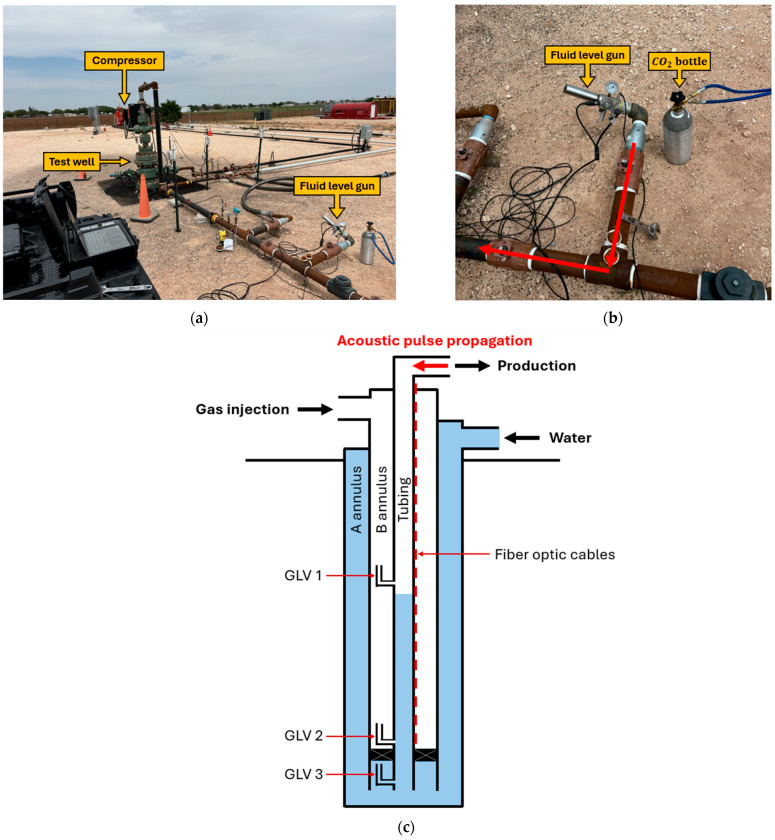
(**a**) Test well connected pipelines and a fluid level gun. (**b**) Fluid level gun connection with arrow indicating pulse propagation direction. (**c**) Schematic cross-section of the test well configuration.

The test well is 430 m deep and consists of 9-5/8 in 36# (244 mm 54 kg/m) casing (A annulus), 5-1/2 in 17# (140 mm 25 kg/m) casing (B annulus), and 2-7/8 in (73 mm) tubing. Two fiber optic cables (one single-mode fiber and one multimode fiber) run 112 m from the DAC to the test well, and then an additional 410 m inside the well. In the well, the fiber optic cables were deployed inside a ¼ in (6.4 mm) stainless steel capillary tube banded to the outside of the 2-7/8 in (73 mm) tubing. The capillary tubing containing the fibers runs parallel to the tubing. Figure 2 shows a schematic of the test well and how a fluid level gun was connected to the flowline to send an acoustic pulse down the tubing. Water is injected into the A annulus and enters the tubing through the third electric gas lift valve (GLV 3). A packer and a blanking plug at the bottom of the tubing prevent water from entering the tubing from locations other than GLV 3. All gas lift valves in the well are surface-controlled gas lift valves with a maximum port size of 3/8 in (9.5 mm). A controlled interface at surface allows user-input commands to control an electric motor in each valve to control its opening and closing. Nitrogen is injected into the B annulus using the compressor and enters the tubing through GLV 2. Each gas lift valve has sensors that measure temperature and pressure in the tubing and the B annulus.

Acoustic pulses were generated using a commercially available air gun connected at the surface. The air gun was charged with compressed nitrogen at a pressure differential maintained between 2.1 and 2.4 MPa. The charging pressure was kept approximately constant for all shots during the experiment to ensure repeatable acoustic excitation.

Two field-scale experiments were performed on 18 March and 25 March 2025 under nominally identical conditions. The geometric and operating conditions of the test well are summarized in Table 1.

A single mode, silica fiber with carbon-polyimide coating was deployed in a ¼" (6.4 mm) stainless steel capillary tube banded to the 2-7/8" (73 mm) tubing for the DAS measurements as mentioned earlier. Key specifications of the deployed cable are summarized in Table 2.

The DAS interrogator used in this study was a Sintela Onyx unit (Sintela Ltd., Bristol, UK). The main acquisition parameters are summarized in Table 3 and were identical for both tests. These settings define the spatial and temporal resolution of the DAS data and were kept constant across all tests.

Depth referencing of the DAS data was established through a channel-mapping procedure. The DAS interrogator records acoustic response along the total fiber length referenced to the interrogator location. To identify the physical wellhead position along the fiber, a surface hammer impact test was conducted at the wellhead prior to the experiments. The hammer strike generated a strong impulsive acoustic signal that was clearly observed in the DAS record. This response corresponded to a fiber distance of approximately 112 m from the interrogator. This location was therefore defined as the wellhead reference point (0 m depth). All DAS waterfall plots and depth-based analyses presented in this study are referenced to this calibrated origin.

**Figure 3:**
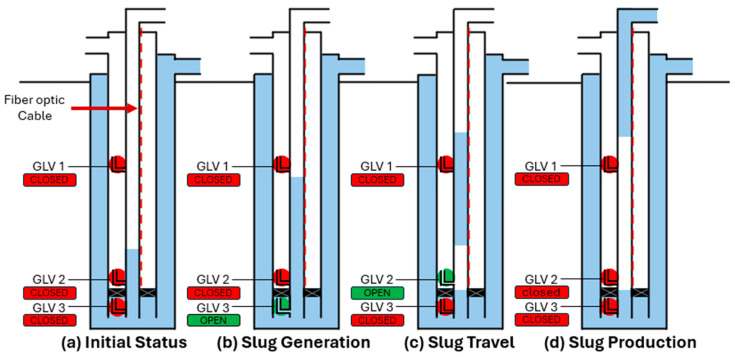
Sequential schematics of the slug generation and production process: (**a**) Initial state with all GLVs closed. (**b**) Water fed into the tubing through GLV 3. (**c**) Slug generated by gas injection through GLV 2 after closing GLV 3. (**d**) Slug reaching the surface.

To run an experiment, all the gas lift valves are initially closed (Figure 3a). We feed water from the A annulus into the tubing through GLV 3 to create a liquid slug of predetermined length, as shown in Figure 3b. We verify the slug length using the pressure sensor installed at GLV 2. After the desired slug length is reached, we close GLV 3 and inject nitrogen into the B annulus to pressurize the casing up to a target pressure higher than the tubing pressure. Once the desired casing pressure is achieved, we turn off the compressor to reduce background noise for the fiber optic cable and perform a fluid level shot to confirm the depth of the liquid slug. Then, we open GLV 2 to inject gas underneath the liquid column and allow produce the liquid slug through the tubing as illustrated in Figure 3c. Throughout this process, we continuously monitor the slug movement using DAS. As the slug is traveling up the tubing, we fire acoustic pulses at 10 s intervals using a commercially available air gun (Echometer Company, Wichita Falls, TX, USA). During each shot, the fluid level gun fires a single acoustic pulse into the tubing. Eventually, the slug reaches the surface, as shown in Figure 3d. After the slug is produced at surface, we close GLV 2 to end the experiment.

### Theoretical Background

**Figure 4:**
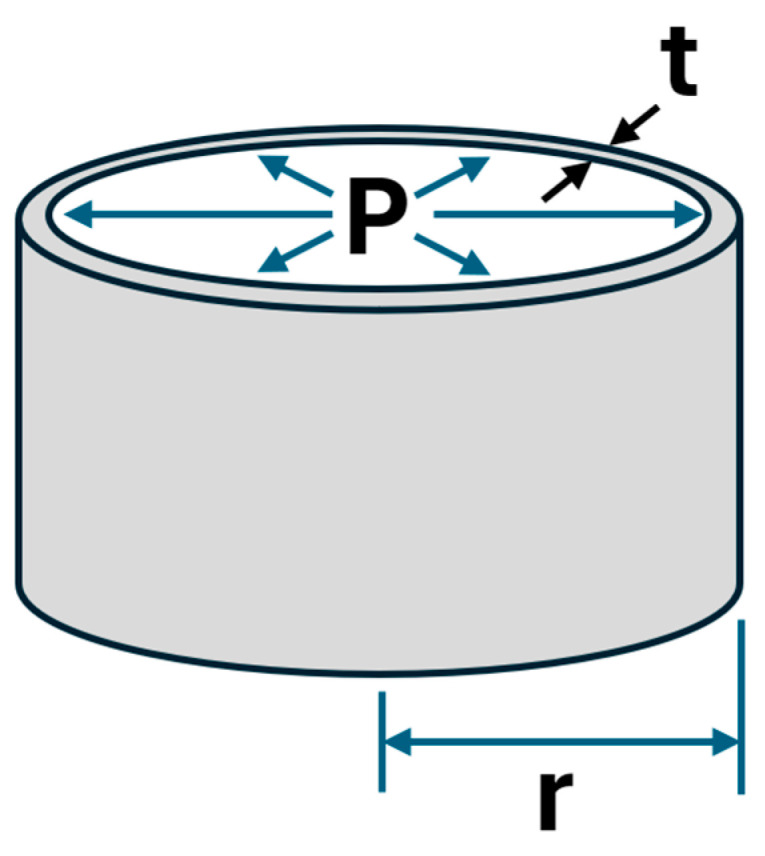
Thin-walled cylinder model showing internal pressure P acting radially on a tube of inner radius r and wall thickness t. This model forms the basis for deriving hoop and axial stresses and the resulting axial strain detected by DAS.

Because DAS detects axial strain changes along the fiber, it responds to local pressure changes in the tubing as an acoustic pulse propagates. The tubing wall can be idealized as a thin-walled cylinder of inner radius r and wall thickness t (t ≪ r), subjected to an internal pressure P, as illustrated in Figure 4.

Under this configuration, the hoop stress is given by

(1) $$\sigma_{h}=\frac{Pr}{t}$$

The axial stress is related to the hoop stress through Poisson’s ratio ν:

(2) $$\sigma_{a}=-\nu \sigma_{h}=-\nu \frac{Pr}{t}$$

Applying Hooke’s law gives the axial strain

(3) $$\varepsilon_{a}=\frac{\sigma_{a}}{E}=-\frac{\nu }{E}\frac{Pr}{t}$$

where E is the Young’s modulus of the tubing steel.

For an incremental pressure perturbation dP, the incremental axial strain is

(4) $$d\varepsilon_{a}=-\frac{\nu r}{E}\frac{dP}{t}$$

These relationships establish the direct proportionality between small pressure changes and the DAS phase response. Equation (4) implies a positive pressure pulse should result in a compressive (negative) strain change. Conversely, a negative pressure pulse should result in a tensile (positive) strain change on the fiber.

**Figure 5:**
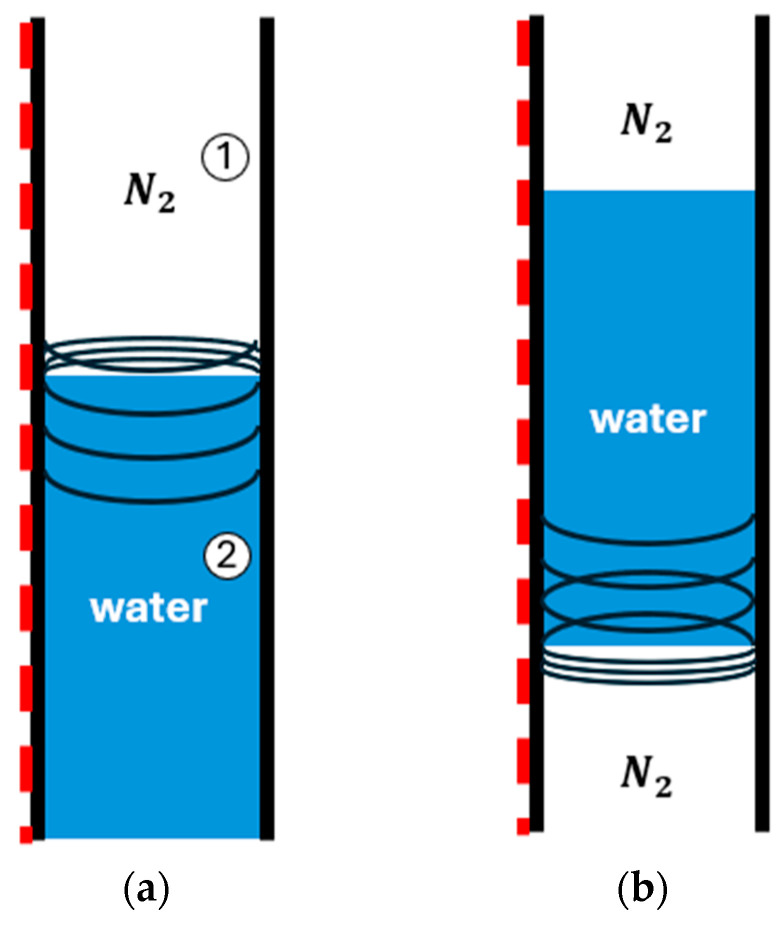
Schematic of nitrogen–water interfaces showing the propagation of the air gun pressure pulse: (**a**) early stage with nitrogen above water; (**b**) fully developed slug with nitrogen above and below, indicating reflection and transmission points. The circled numbers 1 and 2 denote medium 1 (N_2_, impedance z_1_) and medium 2 (water, impedance z_2_), respectively.

Consider what happens when an acoustic pressure pulse reaches a nitrogen–water interface inside the tubing. Figure 5 is a depiction of the behavior of an acoustic pressure pulse in a nitrogen–water interface. Acoustic impedance contrasts calculated from Table 4 yield reflection and transmission coefficients $\tilde{R}$ and $\tilde{T}$ [26].

(5) $$\tilde{R}=\frac{z_{2}-z_{1}}{z_{2}+z_{1}}\;\;\;\;\;\cdots \;\;\;\;\;reflection\;coefficient$$

(6) $$\tilde{T}=\frac{{2z}_{2}}{z_{2}+z_{1}}\;\;\;\;\;\cdots \;\;\;\;\;transmission\;ceofficient$$

Using the measured properties (Table 4), we obtain the following for the nitrogen–water interface.

(7) $$\tilde{R}=\frac{z_{2}-z_{1}}{z_{2}+z_{1}}=0.996$$

(8) $$\tilde{T}=\frac{2z_{2}}{z_{2}+z_{1}}=1.996$$

The amplitude of the reflected wave is 99.6% of the initial value, whereas the amplitude of the transmitted wave in the liquid is nearly twice the initial value. It is worth noting that the acoustic wave speed c used in the impedance definition z=ρc can be influenced not only by the bulk fluid properties but also by the elasticity of the tubing wall. In elastic tubes, the propagation velocity of pressure waves may be reduced relative to the bulk sound speed due to radial deformation of the pipe wall, as classically described by the Moens–Korteweg formulation for waves in elastic tubes [27,28]. This dependence involves both fluid and structural parameters (e.g., fluid bulk modulus and pipe diameter, wall thickness, and Young’s modulus). In the present work, we use the bulk fluid sound speed for the impedance estimates for simplicity and consistency with conventional acoustic fluid level analysis; this approximation does not change the qualitative conclusion that the gas–liquid impedance contrast is large and dominates the reflected and transmitted amplitude behavior at the interface.

**Table 4:** Acoustic properties of nitrogen and water used for reflection and transmission calculations [29].

	ρ (kg/m^3^)	c (m/s)	z (kg/m^2^·s)
Nitrogen (at 690 kPa, 21 °C)	7.9	354	2.8 × 10^3^
Water	1000	1485	1.49 × 10^6^

When the propagating pulse encounters a liquid slug, the reflected amplitude remains nearly unchanged but reverses polarity ($\tilde{R}$ = −0.996), and only a negligible fraction of acoustic energy ($\tilde{T}$ = 0.004) is transmitted below the slug. These coefficients indicate difficulty for the acoustic pulse to transmit below the liquid slug. The wave oscillates between compressive strain (negative phase shifts) and tensile strain (positive phase shifts) within the liquid slug.

The DAS response is controlled by both the elastic response of the tubing and the impedance contrast at the moving gas–liquid interface. A thin-walled cylinder model relates internal pressure to axial strain along the tubing wall, so that compressive strain corresponds to pressure increases and tensile strain to pressure decreases. The fluid level gun generates an acoustic pulse that travels down the liquid column and reflects at the nitrogen–water interface, where the large difference in acoustic impedance creates a strong negative reflection coefficient. Under static conditions, repeated reflections form a partially trapped reverberation field in the liquid column, visible as V-shaped patterns in the DAS derivative waterfall plots. During slug ascent, the top and bottom interfaces of the slug act as moving reflection boundaries, and the intersections of downward and upward sloping events mark their instantaneous positions.

## 3. Results

This section shows the experimental results obtained from DAS measurements. The results are first shown for the reference case without an acoustic pulse, prior to introducing the air gun.

### 3.1. Results—No Acoustic Pulse

**Figure 6:**
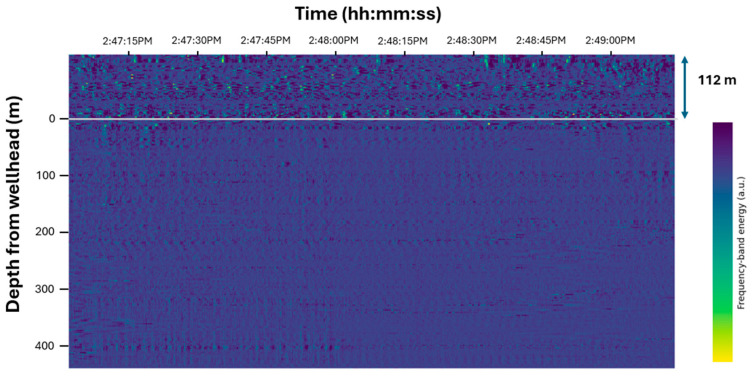
DAS waterfall plot recorded during slug production, showing no distinct acoustic reflections associated with slug movement. This indicates that passive DAS, under these field conditions, did not reveal a clear slug-related signal. The colorbar represents FBE expressed in arbitrary units (a.u.).

Before introducing the fluid level gun, we acquired DAS data during nitrogen-driven slug production without any external acoustic excitation. Under these conditions, the distributed acoustic record showed no distinct reflections or coherent patterns associated with slug movement. Figure 6 shows a frequency band energy (FBE) plot of the acoustic energy at 0.1 s intervals during the slug production over the frequency ranges 0 to 500 Hz. The fiber has 112 m of surface length before entering the wellhead. Below the wellhead, there are no observable signals related to liquid slug production. This result indicates that, at the prevailing noise level and fiber-to-tubing coupling, passively listening to slug movement using DAS does not provide adequate signal for reliable slug characterization. Consequently, an external acoustic source was required to generate a high-contrast reflection pattern for reliable slug tracking.

### 3.2. Results—Acoustic Pulse with Static Liquid Column

**Figure 7:**
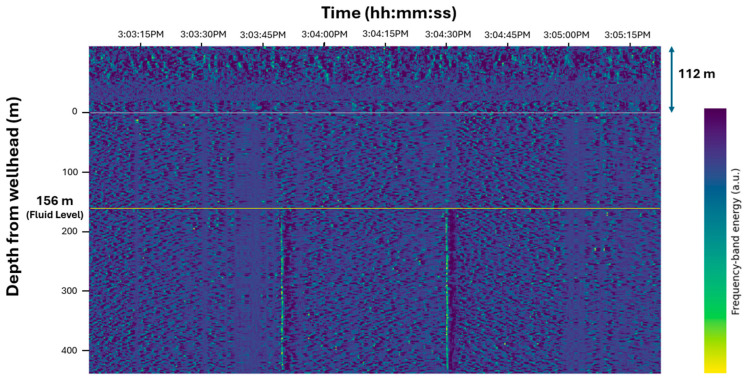
Fluid level visualized on the FBE plot before opening GLV 2. The colorbar represents FBE expressed in arbitrary units (a.u.).

We then observed the DAS response to an acoustic pulse fired with a static liquid column in the wellbore. The fluid level was determined to be 155 m from the two-way travel time of the acoustic pressure pulse measured by a surface microphone, and 156 m from the DAS-based FBE plot, as shown in Figure 7. The depth discrepancy was 0.7%. This close agreement between two independent methods confirms that the FBE reflection band accurately represents the gas–liquid interface at the start of the test and supports the use of DAS as a distributed fluid level measurement.

**Figure 8:**
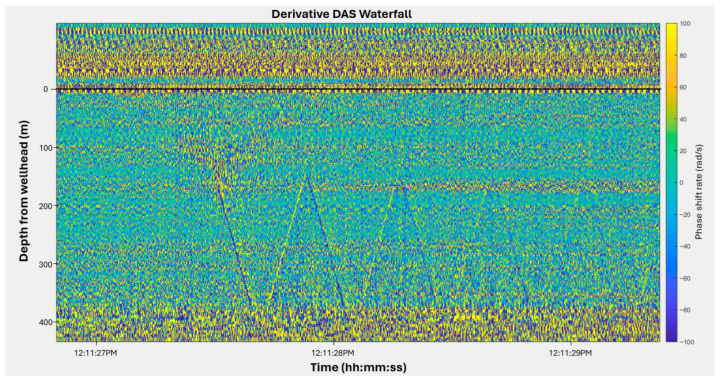
Full bandwidth phase shift derivative waterfall plot showing acoustic pulse propagation and repeated reflection within a static water column. The colorbar indicates phase shift rate in rad/s.

Figure 8 shows the full-bandwidth derivate waterfall plot recorded in a static water column. The downgoing acoustic pulse generated by the gas gun transmits from the nitrogen to the water reflected at the liquid surface. The acoustic pulse can be seen repeatedly reflected within the liquid column. The incident and reflected pulses intersect in the time-depth domain, producing characteristic V-shaped patterns that indicate the instantaneous position of the liquid surface. Each repetition of this pattern demonstrates that the acoustic pulse energy is largely trapped within the water column due to the strong impedance contrast at the interface. This static example illustrates the physical basis of the boundary-identification method applied later, where moving interfaces produce similar V-shaped intersections in DAS data. In addition, the reflected signal exhibits a phase inversion relative to the incident pulse, consistent with reflection from a lower-impedance interface such as the nitrogen–water boundary. This polarity reversal agrees with the theoretical background, where compressive and tensile phases alternate as the acoustic pulse reflects between the gas and liquid layers. Because the geometry and timing of these reflections remain constant in static condition, this pattern serves as a calibration reference for subsequent slug flow experiments. Deviations from this baseline in later figures can therefore be directly attributed to interface motion or changes in acoustic coupling during dynamic flow.

Based on the static-liquid response described above, slug boundaries during dynamic flow were identified using the same reflection principles. In the derivative waterfall and FBE representations, moving gas–liquid interfaces generate V-shaped intersections formed by the interaction of downward- and upward-propagating acoustic pulses. These intersections define the instantaneous locations of the slug interfaces in the depth–time domain.

The lower interface is generally more clearly defined because it can be identified from the intersection between the first downgoing pulse and the first upgoing reflected pulse, which forms a distinct and repeatable V-shaped pattern. This reflection geometry is enabled by the strong acoustic impedance contrast at the gas–liquid interface, which traps acoustic energy within the liquid column and ensures coherent pulse interactions.

In contrast, the upper interface may exhibit weaker or more diffuse intersection patterns, particularly in aerated regions or during later stages of slug ascent. In such cases, boundary localization carries increased positional uncertainty.

This uncertainty primarily affects instantaneous slug length estimation, which relies on the spatial separation between the identified upper and lower interfaces. The resulting uncertainty is typically limited to several DAS channels and does not alter the overall temporal evolution of slug growth and collapse. Slug velocity estimation is comparatively less sensitive to boundary ambiguity, as velocity is derived from the slope of the boundary trajectory over multiple acoustic shots rather than from a single spatial measurement. As a result, interface uncertainty has minimal influence on velocity trends reported in this study.

### 3.3. Results—Moving Liquid Slug with Multiple Acoustic Pulses

**Figure 9:**
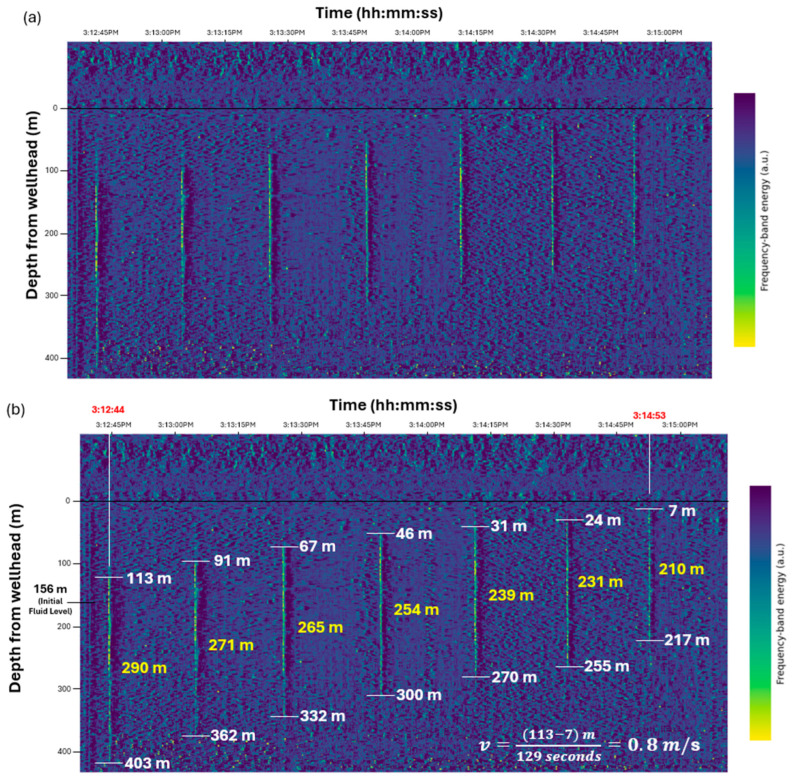
DAS response to a slug rising in the tubing. (**a**) FBE plot of slug rise. (**b**) Annotated FBE plot showing slug velocity and length. The red timestamps indicate the first and last observed slug events used to calculate the average slug velocity. The colorbar represents FBE expressed in arbitrary units (a.u.).

Figure 9 shows the DAS response to a liquid slug rising in the tubing after GLV 2 was opened. Figure 9a presents the FBE plot during slug rise, and Figure 9b shows the annotated plot. From the upward movement of the slug top surface, the average slug velocity was calculated to be 0.8 m/s. The slug length decreased from 290 m at the initial GLV 2 opening to 210 m by the time the slug reached the surface, reflecting liquid fallback. These FBE plots provide an overview of slug velocity and length evolution.

**Figure 10:**
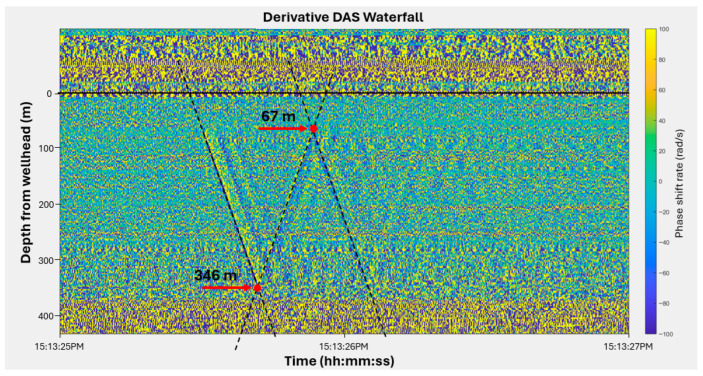
Derivative waterfall plot showing the identification of slug boundaries during upward motion. The V-shaped intersections define slug boundaries, and their vertical separation corresponds to slug length. The dotted lines indicate the trajectories used to identify the V-shaped intersection points. The colorbar indicates phase shift rate in rad/s.

Figure 10 shows a waterfall plot of the time derivative of the accumulated DAS phase shift. This plot is a useful diagnostic plot to determine the slug boundaries. The bottom boundary of the liquid slug was consistently identified from the V-shaped intersection formed by the first downgoing pulse and first upgoing reflection. The top boundary sometimes did not exhibit as clear a response as the bottom boundary. When the second downgoing reflection was sufficiently coherent, the top boundary was identified using its intersection with the first upgoing reflection. When this reflection became too weak or diffuse to form a reliable intersection, the top boundary was estimated from the onset of the first downgoing event instead. The vertical separation between the identified top and bottom interfaces represents the instantaneous slug length. Because the top boundary was sometimes determined from weaker or less coherent reflections, its depth carries greater uncertainty than that of the bottom interface.

**Figure 11:**
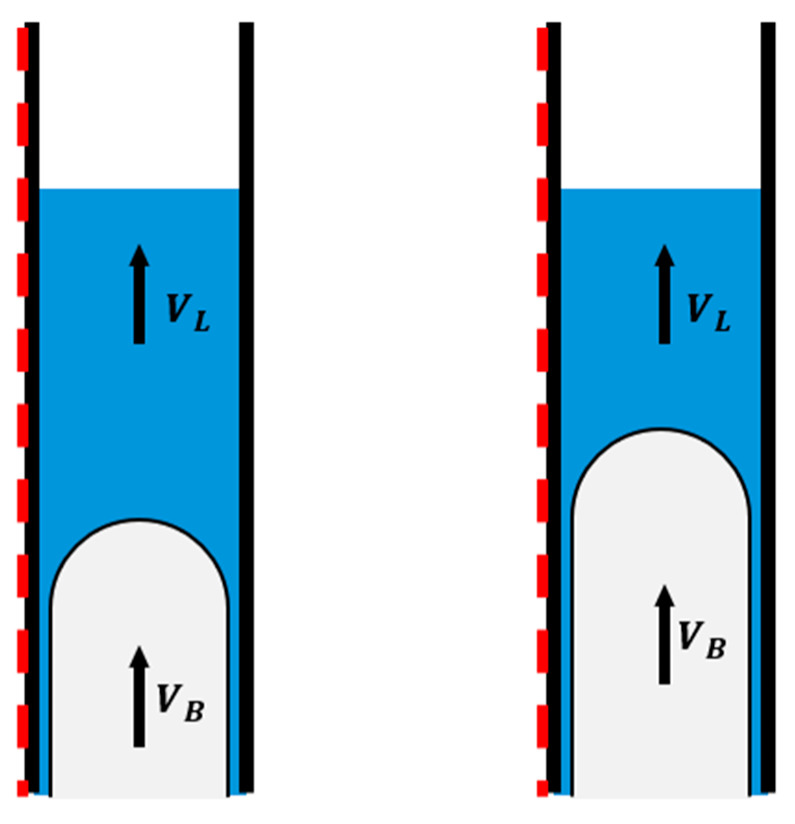
Conceptual schematic showing top (V_L_) and bottom (V_B_) boundary velocities of a rising liquid slug. The arrows indicate the upward flow direction and boundary velocities, while the red dotted line represents the fiber optic cable installed outside the tubing.

The resulting depth–time intersection analysis produced estimates of slug length and velocity for each test. Results for both experimental sequences are included in Table 5 and Table 6. Figure 11 illustrates the definitions of the upward liquid surface velocity V_L_ and the gas bubble front velocity V_B_. The bubble front marks the interface where expanding gas displaces the liquid column, whereas the top boundary corresponds to the upward movement of the liquid slug surface. In several shots, the bottom boundary could not be reliably identified because the downgoing reflection weakened significantly. In a few cases, the shot timestamp was also unavailable. For these shots, the slug length and velocity could not be calculated, and the corresponding entries in Table 5 and Table 6 are left blank.

For both experiments, the estimated slug length decreased progressively with each successive air gun shot. When the measurements are referenced to the GLV 2 opening time, this trend indicates a gradual reduction in liquid holdup as the slug propagates upward along the tubing.

**Figure 12:**
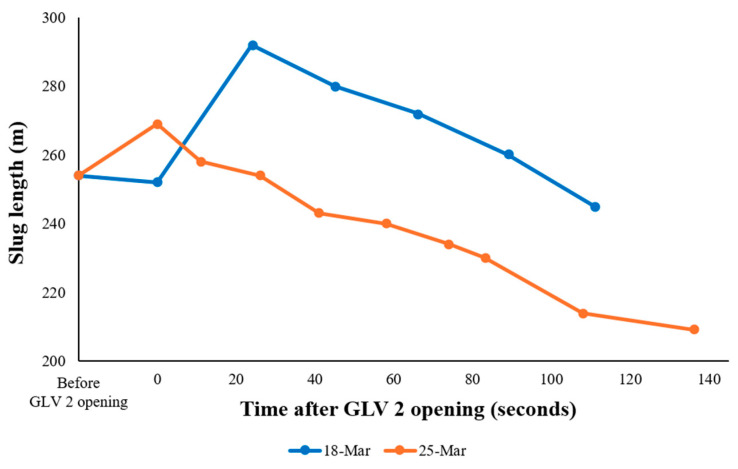
Slug evolution for 18 March and 25 March experiments. Time is referenced to the instant when GLV 2 opens.

Figure 12 presents the evolution of slug length for both test dates plotted as a function of elapsed time since GLV 2 opening. Following valve activation, an initial increase in slug length is observed in the 18 March experiment during the early injection stage. This behavior reflects a startup transient associated with the gradual opening of the gas-lift valve, during which the injected gas was insufficient to lift the liquid column and instead accumulated within the tubing, resulting in an apparent elongation of the slug as detected by DAS.

As gas–liquid separation became established, gravitational liquid fallback emerged as the dominant mechanism governing slug evolution. Under these conditions, a monotonic decrease in slug length is observed for both experiments, demonstrating consistent slug-drainage behavior once stable injection conditions are reached.

The evolution of slug length during the experiments further highlights the transient character of the generated slugs. In fully developed vertical slug flow, repeated slugs continually shed liquid from the tail while simultaneously picking up liquid at the bubble front. This exchange produces a nearly constant equilibrium length, typically around 30–35 pipe diameters, as reported in classical studies [30,31]. In contrast, the single slug tests performed here did not include continuous replenishment mechanisms. With no subsequent slugs following the initial injection, the ascending slug progressively lost liquid due to gravitational drainage, resulting in steadily decreasing slug length. Overall, these observations confirm that the experiments captured a true transient slug process rather than a steady-state slug flow. The DAS measurements were sufficiently sensitive to track these behaviors, including bubble-liquid velocity differences and the progressive drainage that shaped the evolving slug geometry.

**Figure 13:**
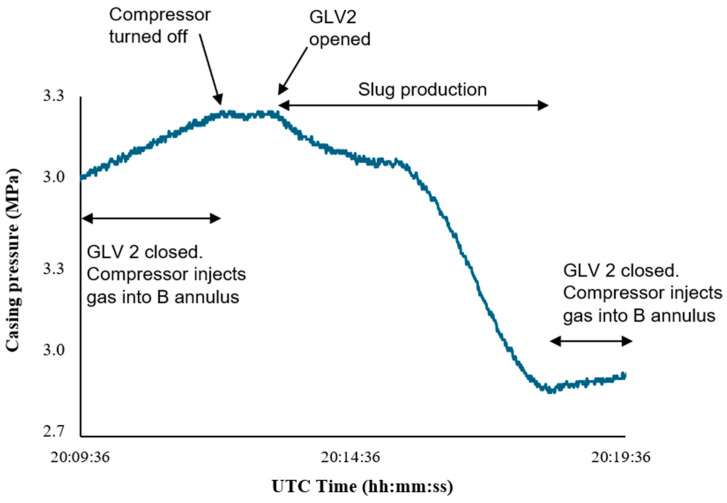
Casing pressure change during 18 March.

**Figure 14:**
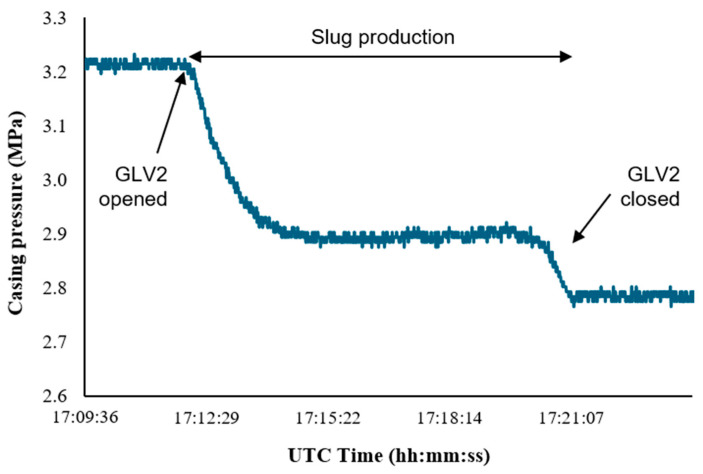
Casing pressure change during 25 March.

The casing pressure histories for both experiments (Figure 13 and Figure 14) show a sustained pressure decline when GLV2 is opened, indicating continuous gas transfer from Annulus B into the tubing during slug production.

We can extract the gas injection rate from the casing pressure histories shown in Figure 13 and Figure 14. The real gas law was used in deriving an equation for the gas injection rate in MSCFD, as shown in Equation (9).

(9) $$q_{g(t)}=\frac{V_{ann}T_{sc}}{P_{sc}T_{avg}z}\cdot \frac{{dP}_{c}}{dt}\cdot \frac{1440}{1000}$$

where $V_{ann}$ is the annular volume, $T_{sc}$ and $P_{sc}$ is the temperature and pressure at standard conditions, respectively, z is the compressibility factor, $T_{avg}$ is the average temperature and ${dP}_{c}$/dt is the rate of change in casing pressure with respect to time in psi/min.

The casing volume was calculated using

(10) $$A_{ann}=\frac{\pi }{4}({ID}_{casing}^{2}-{OD}_{tubing}^{2})$$

(11) $$V_{ann}=A_{ann}L$$

The average temperature was obtained by calculating the mean of the temperature at the surface and the temperature of the casing. The gas compressibility factor, z, was determined using the Viral Equation of State

(12) $$z=1+\frac{{BP}_{c}}{RT}$$

where the second virial coefficient B(@$T_{avg}$) = −3.6858.

**Figure 15:**
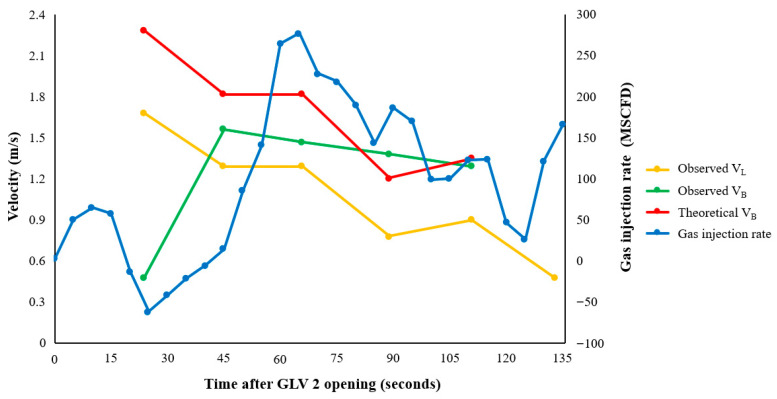
Comparison of experimental and theoretical slug and bubble velocities for 18 March experiment. Time is referenced to the instant when GLV 2 opens.

**Figure 16:**
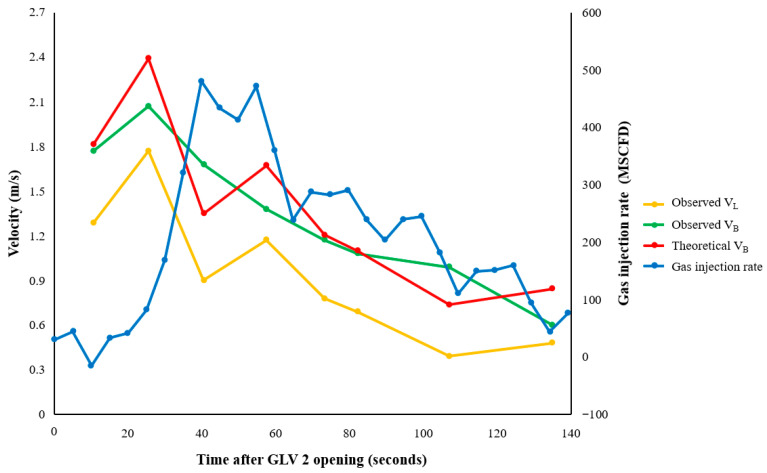
Comparison of experimental and theoretical slug and bubble velocities for 25 March experiment. Time is referenced to the instant when GLV 2 opens.

Figure 15 and Figure 16 compare the calculated gas injection rate for each experiment with the slug and bubble velocities determined from the DAS waterfall plots. The boundary velocities consistently showed V_B_ > V_L_ in both experiments. This behavior is consistent with the fundamental physics of Taylor bubble motion in vertical conduits: the gas bubble expands and migrates upward faster than the liquid phase it displaces.

To interpret the observed bubble velocities, the classical vertical slug flow drift-flux correlation was applied [32]:

(13) $$V_{B}=C_{o}V_{L}+v_{o}^{*}\sqrt{gd}$$

where $C_{o}$ is the distribution coefficient, $v_{o}^{*}$ is the drift velocity parameter, g is gravitational acceleration expressed in m/s^2^, and d is the tubing inner diameter in meters. Parameters of $C_{o}$ = 1.2 and $v_{o}^{*}$ = 0.35 were adopted following established correlations for steady vertical slug flow [32]. Comparison of the observed velocities with the theoretical trend (shown in Figure 15 and Figure 16) reveals close agreement. Bubble velocities remained within approximately ±13% of the predicted curve during the 18 March tests and within ±20% during the 25 March sequence. Both boundary velocities gradually decreased throughout the experiment due to Annulus B pressure depletion and decreased gas injection rates. The estimated injection rate did not remain constant during the experiment but varied continuously as annulus pressure was depleted. As one illustrative example, the inferred injection rate for the 18 March test decreased from approximately 181 MSCFD (5130 m^3^/day) during the early stage to about 166 MSCFD (4700 m^3^/day) at an intermediate stage, and further to roughly 84 MSCFD (2380 m^3^/day) toward the late stage of the experiment. This time-varying gas supply is consistent with the reduction in the upper and lower slug boundary velocities shown in Figure 15 and Figure 16. This depletion implies a dynamically evolving gas injection rate rather than a steady injection condition, providing the driving context for the observed gradual decrease in both bubble and slug boundary velocities shown in Figure 15 and Figure 16.

## 4. Discussion

### 4.1. Measurement Uncertainty

Regarding measurement uncertainty, the spatial resolution of the DAS measurement is governed by the 3.2 m gauge length and the 1.6 m channel spacing, which together define the effective averaging interval along the tubing. Slug boundary depths are identified from the intersections of downward and upward sloping events in the derivative waterfall and FBE representations. As a result, the depth uncertainty is typically on the order of one to two channels (1.6–3.2 m), depending on the sharpness of the V-shaped reflection pattern. In time, the 2 kHz acquisition rate provides sub-millisecond sampling, while the FBE computation, with a 0.5 s averaging window to improve signal-to-noise ratio, yields an effective temporal resolution of approximately 0.5 s for FBE-based boundary tracking. When combined, these spatial and temporal resolution limits produce velocity uncertainties on the order of a few percentage points.

### 4.2. No Observable Signal Above Noise Floor Without Acoustic Pulse

Previous DAS studies on slug flow detection were mostly conducted in laboratory flow-loop facilities under well-controlled acoustic conditions [21,33]. In contrast, the present experiment was performed in a field-scale test well, where unavoidable environmental noise, such as pump vibration, surface equipment, and pressure fluctuations due to flow through the gas lift valve, likely masked the subtle acoustic and thermal signals generated solely by the rising liquid slug. This difference between laboratory and field conditions may partly explain why the signals were not clearly resolved in our test well without application of the air gun. Because the slug’s self-generated noise was below the ambient noise floor, separating passive slug signature from the background was not feasible. This does not contradict previous laboratory findings, as the present field well exhibits higher noise levels and weaker coupling than concentric laboratory fiber wraps.

In contrast to passive DAS measurements, the application of externally generated acoustic pulses introduces a controlled and repeatable source with known timing, frequency content, and amplitude. These pulse characteristics significantly enhance sensitivity to acoustic impedance contrasts at gas–liquid interfaces, allowing reflected wavefields to be distinguished from background noise even under field-scale conditions. As a result, the visibility of slug boundaries was found to be strongly dependent on the presence and characteristics of the acoustic pulse, explaining why passive DAS alone was insufficient for reliable slug detection in the present experiments.

### 4.3. No Observable Signal in the Nitrogen-Filled Portion of the Tubing

A measurable DAS response to the air gun acoustic pulse is observed primarily in the liquid-filled portion of the wellbore, whereas no clear response is detected in the nitrogen-filled interval in either the FBE or derivative waterfall representations.

The DAS response is governed by tubing axial strain induced by transient pressure perturbations. As introduced in Equation (4), the incremental axial strain scales linearly with the local pressure fluctuation ($d\varepsilon_{a}$∝dP) [15].

For an acoustic disturbance, the local pressure fluctuation dP may be approximated by the acoustic pressure perturbation p’, which for a one-dimensional wave satisfies $p^{\prime }=\rho cu$, where ρ is the fluid density, c is the acoustic velocity, and u is the particle velocity [26]. Accordingly, the expected pressure amplitude ratio between liquid- and gas-filled sections can be estimated as

(14) $$\frac{{dP}_{liq}}{{dP}_{gas}}\approx \frac{{\left(\rho c\right)}_{liq}}{{\left(\rho c\right)}_{gas}}$$

Using representative fluid properties listed in Table 4, this order-of-magnitude estimate yields a pressure amplitude ratio on the order of two. This modest difference indicates that pressure perturbation alone is unlikely to be the dominant mechanism responsible for the observed DAS amplitude contrast, although it may contribute as a secondary factor.

In contrast, wavelength-related effects play a more significant role. In the nitrogen-filled interval, the acoustic wavelength is comparable to the applied 3.2 m DAS gauge length, leading to spatial averaging and partial cancelation of strain within each gauge section. The combined effects of weak pressure–strain coupling in gas and gauge length averaging therefore provide a consistent explanation for the absence of a clearly observable DAS response in the nitrogen-filled portion of the tubing.

To evaluate whether spatial averaging may further suppress the measured response in the nitrogen section, we next consider the spatial resolution of the DAS measurement relative to the acoustic wavelength in nitrogen, compared with the wavelength in water. Because the acoustic frequency ƒ remains approximately constant as the pulse crosses the nitrogen–water interface, the local wavelength satisfies

(15) $$\lambda =\frac{c}{f}$$

From this relation, the water and nitrogen wavelengths are

(16) $$\lambda_{w}=\frac{c_{w}}{f}$$

(17) $$\lambda_{N}=\frac{c_{N}}{f}$$

and the ratio is

(18) $$\lambda_{w}=\frac{c_{N}}{c_{w}}=0.238,\;or\;\sim 25\%$$

The dominant frequency ƒ used in these estimates is obtained directly from the DAS waveform by measuring the time interval Δt between successive valley and peak points,

(19) $$f=\frac{1}{2\Delta t}$$

**Figure 17:**
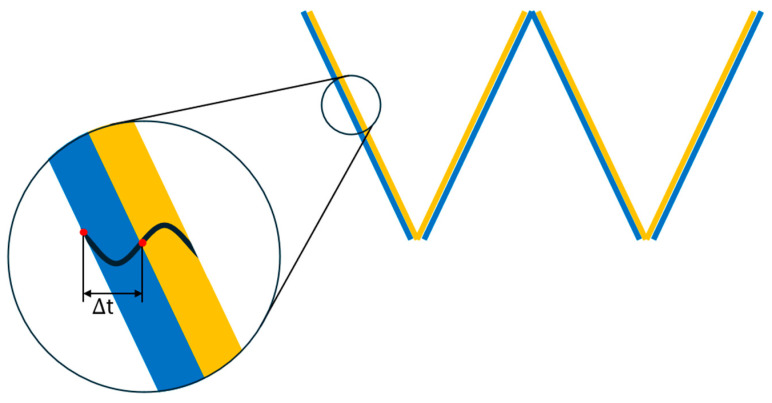
Simplified representation of a DAS waveform segment extracted from the derivative waterfall, used to determine Δt and calculate the dominant frequency ƒ. The blue and yellow bands represent alternating acoustic signal segments from which the half period (Δt) is measured. Insert shows valley–peak measurement for Δt.

These relationships show that the nitrogen wavelength is much shorter than the water wavelength. Figure 17 is schematic of the DAS waveform segment that is used to determine Δt and calculate the dominant frequency ƒ. The inset shows the valley–peak measurement used for Δt.

To further quantify the dominant frequency, we additionally measured half periods (Δt) using the blue/yellow band widths, as described below. The dominant frequency of the acoustic signal in the nitrogen segment was estimated from the derivative waterfall plot. In this representation, the acoustic pulse produces alternating blue and yellow bands, which repeat periodically along the time axis. The width of blue, the width of yellow, and the mid-blue to mid-yellow spacing all correspond to half of the acoustic period (Δt). By measuring these intervals in units of samples and converting them into time, the frequency can be obtained.

With a sampling rate of 2000 Hz, one count on the time axis corresponds to 0.0005 s. Thus, for a measured width N (in samples), the half period (Δt) is

(20) $$∆t=\frac{N}{2000}\;s$$

and the dominant frequency is determined as

(21) $$f=\frac{1}{2∆t}$$

Multiple measurements of the width of the blue (compressive) portion of a pulse were taken from the derivative waterfall plot. The measured half periods fall in the range of

(22) 0.007 s ≤2∆t≤0.014 s

and the corresponding frequency is

(23) $$f=\frac{1}{2\;∆t}$$

which yields

(24) 71.4 Hz ≤f≤142.8 Hz

Using the representative frequency of 107 Hz and acoustic velocity of $C_{N_{2}}$= 354 m/s in nitrogen, the wavelength is estimated as

(25) $$C_{N_{2}}=\lambda_{N_{2}}f$$

(26) $$\lambda_{N_{2}}=\frac{C_{N_{2}}}{f}=\frac{354\;m/s}{107\;Hz}=3.3\;m$$

For DAS to effectively resolve the acoustic wave, the gauge length $L_{g}$ must be less than a quarter of the wavelength:

(27) $$L_{g}<\frac{\lambda }{4}\approx 0.8\;m$$

However, the present field configuration used a 3.2 m gauge length, which is significantly larger than the quarter-wavelength threshold. Under these conditions, positive and negative strain within each gauge section can spatially average and partially cancel, attenuating the recorded signal. The relatively long gauge length may reduce sensitivity to short wavelength acoustic components, particularly in gas-filled sections.

Finally, gauge length averaging alone may not fully explain the absence of a measurable DAS response in the nitrogen-filled interval. Based on the strain–pressure relationship in Equation (4), the axial strain amplitude scales linearly with the local pressure fluctuation. Using the estimated pressure–amplitude ratio between water and nitrogen, the anticipated strain response in nitrogen is therefore approximately half of the observed in the liquid-filled section.

When this scaling is applied to the measured DAS phase amplitudes, the resulting phase response in nitrogen is estimated to be comparable to, or below, the measured DAS noise floor determined from baseline data. This quantitative comparison indicates that the expected acoustic response in nitrogen is likely obscured by system noise, even in the absence of spatial averaging effects.

## 5. Conclusions

In our experimental study, we could not readily detect the location of a large liquid slug in a multiphase fluid based on turbulent fluid noise alone. We required an external noise source (in this case, an acoustic pulse generated by a fluid level gun) to consistently observe the slug. The acoustic pulse generates more noise for the fiber optic cable over sections of the tubing covered by the liquid slug. This allows us to see not only the top of the slug, but the bottom as well, as it is produced. We demonstrated a method combining DAS with fluid level shots to detect the length of a liquid slug as it is produced.

This approach may support improved situational awareness in wells where liquid accumulation affects production performance. By diagnosing the length and velocity of liquid slugs from surface measurements, offshore operators can actively monitor producing wells for severe slugging. Such information could help operators make more informed flow management decisions. While the present work focuses on a vertical test well, similar principles may be explored in other well configurations if supported by future studies.

Several limitations were found during the experiment. First, when the liquid slug is close to the gas gun, it becomes hard to observe the acoustic reflections on the DAS plot. This is likely because of interference near the gun. Second, while the top of the slug appears clearly, the bottom part is sometimes weak or unclear. Lastly, the gas gun we used reloads slowly, limiting the time between shots to 10 s intervals. Because of this, it is hard to see how the slug speeds up in the early stage. In future work, we can improve signal quality by reducing noise, using faster acoustic pulse sources, or combining DAS with other methods.

Learnings from this study include the following:

- When combined with controlled acoustic pulses, DAS successfully identified the location, velocity, and effective length of rising liquid slugs in a vertical well under field-scale conditions.
- The distributed nature of DAS provides continuous spatial coverage along the tubing, enabling detailed tracking of transient multiphase behavior.
- The method demonstrated here offers a practical approach for monitoring liquid accumulations in wells and may assist in broader flow management and diagnostic applications.
- DAS did not record a measurable response to acoustic wave propagation in the nitrogen-filled section due to the spatial resolution of the DAS relative to the wavelength.

## Figures and Tables

**Table 1:** Well configuration and operating parameters used for the tests.

Parameter	Value	Unit
Tubing ID	2.441 (62)	in (mm)
Tubing OD	2.875 (73)	in (mm)
Injection line ID	2.000 (50.8)	in (mm)
Casing ID	4.892 (124)	in (mm)
Packer set depth	1381 (421)	ft (m)
Valve depth (GLV 2)	1345 (410)	ft (m)
Injection line length	98 (30)	ft (m)
Temperature at GLV2	76 (24)	°F (°C)
Tubing pressure at GLV2 before opening the valve	394 (2.72) (18 March) 404 (2.79) (25 March)	psi (MPa)
Surface temperature (T_surf_)	70 (21)	°F (°C)
Annulus pressure at GLV2	462 (3.19) (18 March) 464 (3.20) (25 March)	psi (MPa)
Wellhead pressure	41 (0.28) (18 March) 50 (0.34) (25 March)	psi (MPa)

**Table 2:** Optical fiber specifications of the deployed cable.

Parameter	Value
Core material	Silica
Optical mode	Single mode
Core diameter (µm)	9
Cladding diameter (µm)	125
Coating type	Carbon/Polyimide
Maximum operating temperature	300 °C
Attenuation (dB/km)	~2.6

**Table 3:** DAS interrogator and acquisition parameters.

Parameter	Value	Unit
Sampling rate	2000	Hz
Channel length	1.6	m
Gauge length	3.2	m

**Table 5:** Estimated slug length and velocity from intersection point analysis for the 18 March experiment.

	GLV2 Open	1	2	3	4	5	6	7
Seconds since GLV2 opening (s)	0	24	45	66	89	111	133	153
Top of liquid (m)	164	123	95	68	51	30	19	19
Bottom of liquid (m)	416	405	371	339	306	276	-	-
Estimated slug length (m)	252	282	276	271	255	246	-	-
Slug velocity (V_L_) (m/s)	-	1.7	1.3	1.3	0.8	0.9	0.5	-
Bubble velocity (V_B_) (m/s)	-	0.5	1.6	1.5	1.4	1.3	-	-

**Table 6:** Estimated slug length and velocity from intersection point analysis for the 25 March experiment.

	GLV2 Open	1	2	3	4	5	6	7	8
Seconds since GLV2 opening (s)	0	11	26	41	58	74	83	108	136
Top of liquid (m)	147	123	96	82	61	48	42	32	21
Bottom of liquid (m)	416	381	350	325	301	282	272	246	230
Estimated slug length (m)	269	258	254	243	240	234	230	214	209
Slug velocity (V_L_) (m/s)	-	1.3	1.8	0.9	1.2	0.8	0.7	0.4	0.5
Bubble velocity (V_B_) (m/s)	-	1.8	2.1	1.7	1.4	1.2	1.1	1.0	0.6

## Data Availability

All original findings presented in this study are contained within the article.
